# Radiometric Measurement Comparison Using the Ocean Color Temperature Scanner (OCTS) Visible and Near Infrared Integrating Sphere

**DOI:** 10.6028/jres.102.043

**Published:** 1997

**Authors:** B. Carol Johnson, F. Sakuma, J. J. Butler, S. F. Biggar, J. W. Cooper, J. Ishida, K. Suzuki

**Affiliations:** National Institute of Standards and Technology, Gaithersburg, MD 20899-0001; National Research Laboratory of Metrology, 1-1-4, Umezono, Tsukuba-Shi, Ibaraki, 305 Japan; National Aeronautics and Space Administration, Code 920.1, Goddard Space Flight Center, Greenbelt, MD 20771; University of Arizona, Optical Sciences Center, Meinel Building, P.O. Box 210094, Tucson, AZ 85721-0094; Hughes STX Corporation, Greenbelt, MD 20771; NEC Corporation, Space Systems Division, 4035, Ikebe-cho, Tsuzuki-ku, Yokohama, 224 Japan

**Keywords:** calibration, EOS, integrating sphere, measurement comparison, OCTS, ocean color, SeaWiFS, spectral radiometry, remote sensing, transfer radiometers

## Abstract

As a part of the pre-flight calibration and validation activities for the Ocean Color and Temperature Scanner (OCTS) and the Sea-viewing Wide Field-of-view Sensor (SeaWiFS) ocean color satellite instruments, a radiometric measurement comparison was held in February 1995 at the NEC Corporation in Yokohama, Japan. Researchers from the National Institute of Standards and Technology (NIST), the National Aeronautics and Space Administration/Goddard Space Flight Center (NASA/GSFC), the University of Arizona Optical Sciences Center (UA), and the National Research Laboratory of Metrology (NRLM) in Tsukuba, Japan used their portable radiometers to measure the spectral radiance of the OCTS visible and near-infrared integrating sphere at four radiance levels. These four levels corresponded to the configuration of the OCTS integrating sphere when the calibration coefficients for five of the eight spectral channels, or bands, of the OCTS instrument were determined. The measurements of the four radiometers differed by −2.7 % to 3.9 % when compared to the NEC calibration of the sphere and the overall agreement was within the combined measurement uncertainties. A comparison of the measurements from the participating radiometers also resulted in agreement within the combined measurement uncertainties. These results are encouraging and demonstrate the utility of comparisons using laboratory calibration integrating sphere sources. Other comparisons will focus on instruments that are scheduled for spacecraft in the NASA study of climate change, the Earth Observing System (EOS).

## 1. Introduction

Several ocean color sensors on Earth-observing satellites developed by various national governments will be operational in the near future. The results derived from these sensors will be used to address issues relating to global warming, for example, the ability of the oceans to incorporate atmospheric carbon into organic and inorganic forms. In the United States, the Sea-viewing Wide Field-of-view Sensor^1^ (SeaWiFS) [1] and the Moderate Resolution Imaging Spectroradiometer (MODIS) [2] have been designed to provide ocean color data.^2^ SeaWiFS is a product of the National Aeronautics and Space Administration (NASA) Office of Space and Science Applications and Goddard Space Flight Center (GSFC). SeaWiFS was built by Santa Barbara Remote Sensing (SBRS), a subsidiary of Raytheon,^3^ and Orbital Science Corporation is responsible for the spacecraft and delivery into orbit. SeaWiFS is scheduled for launch on the SeaStar satellite sometime after May 1997. MODIS is also being built by SBRS and is scheduled for launch in 1998 along with four other instruments on the AM-1 spacecraft of the Earth Observing System (EOS). EOS is the main program in NASA’s Mission to Planet Earth, which in turn is part of the U.S. study on climate research [3]. The Japanese Ocean Color and Temperature Scanner (OCTS) [4] and the French Polarization and Directionality of Earth’s Reflectances (POLDER) [5] instruments have begun to make ocean color measurements from space. OCTS and POLDER are two of eight instruments on the Advanced Earth Observing Satellite (ADEOS) spacecraft, which was launched at 10:53 a.m. Japan Standard Time on August 17, 1996 from Tanegashima Space Center. OCTS was built by NEC Corporation and POLDER is the product of the Centre Nationale d’Etudes Spatiales (CNES). ADEOS, which has a 3-year design life, is the responsibility of the National Space Development Agency of Japan (NASDA).

Radiometric satellite instruments such as OCTS, POLDER, SeaWiFS, and MODIS are calibrated in the laboratory before integration onto the spacecraft using known sources of spectral radiance such as integrating spheres and blackbody radiators. Most of the satellite instruments have internal calibration sources, or are designed to make measurements relative to the sun. The responsivity to these *on-board* calibrators is also determined before integration and launch. Once in orbit, these systems are used to track the stability of the sensor. As a further study of the accuracy, precision, and stability of the radiometric performance of any single instrument, the results from two or more similar instruments on the same or different spacecraft are compared to each other. Simultaneous results acquired during field experiments using sensors mounted on land, aircraft, ships, or the ocean are also evaluated. In anticipation of these experiments, this work addresses the accuracy of the pre-flight radiometric calibration of one instrument, OCTS. Measurements by four independent groups of the radiometric source that was used to calibrate OCTS at NEC Corporation before it was integrated onto the spacecraft are compared to a February 1995 calibration of the source by NEC Corporation. Each group used a calibrated radiometer to determine the spectral radiance of the integrating sphere source at selected wavelengths in the interval from 400 nm to 1100 nm. The results of this work are significant because traceability to various national standards laboratories is established.

Previous comparison experiments have been performed. The accuracy of the pre-flight calibration of POLDER and OCTS was studied in March and April 1994, when the two instrument teams used portable radiometers to jointly measure the POLDER integrating sphere in Toulouse, France and the OCTS integrating sphere in Yokohama, Japan. The results of the portable radiometers with the POLDER sphere varied by 6 % or less; for the OCTS sphere, the variation was 5 % or less [6]. Experiments to intercompare the two instruments while in orbit are scheduled for 1997. For the EOS AM-1 spacecraft, the pre-flight calibration of MODIS and the visible and near infrared instrument that is part of the Advanced Spaceborne Thermal Emission and Reflection Radiometer (ASTER) was assessed in two experiments. In February 1995, just prior to the measurements reported in this work, the same group of scientists from NIST, NASA/GSFC, NRLM, and UA measured the integrating sphere source that had been used to calibrate the ASTER Visible and Near-Infrared Radiometer (VNIR) instrument [7], and in August 1996 the same group visited Hughes SBRS and made measurements of the sphere that had been used to calibrate MODIS and SeaWiFS [8]. The SeaWiFS Intercalibration Round-Robin Experiments (SIRREX’s) are an additional example of an ongoing cross-calibration activity. For SIRREX-1 to SIRREX-3, several integrating spheres and standard lamps with diffuse plaques were assembled at a common site and measured by a number of instruments. Each year, the results improved, and at SIRREX-3 the difference between two of the instruments for several spheres under optimal measurement conditions was about 1 % [9].

OCTS is one of two fundamental instruments on ADEOS; the mission objectives are to provide continuous global coverage of the Earth’s surface, ocean, and atmospheres. The orbit is sun-synchronous at an altitude of about 800 km and the orbit node is 10:30 a.m. descending. OCTS has eight spectral bands in the visible and near infrared spectral region, and four bands in the infrared. With a spatial resolution of about 700 m and a swath width of about 1400 km, the same area on the Earth is observed every three days. OCTS can be tilted by up to 20° in order to avoid the specular reflection of the sun off the surface of the ocean. The on-board calibrators for the visible and near infrared spectral region consist of halogen lamps and a quasi-telecentric telescope that illuminates the entrance pupil of OCTS with a portion of the solar flux. NASDA is providing the U.S. OCTS science team with the data that correspond to observations of U.S. coastal areas. The center wavelengths, bandwidths, and magnitude of spectral radiance for the pre-flight calibration at NEC Corporation in the visible and near infrared region are given in Table 1. The wavelengths and bandwidths refer to the original specifications for OCTS; the measured values were slightly different. The spectral radiance values for the pre-flight calibration were determined from typical values for ocean and land.

## 2. Instruments

### 2.1 OCTS Integrating Sphere

Table 2a gives the specifications for the ADEOS OCTS integrating sphere; it was built based on experience with the smaller integrating sphere that was used to calibrate the Optical Sensor (OPS) instrument deployed on the Japanese Earth Resources Satellite-1 (JERS-1), which was launched in 1992 [10].

The OCTS sphere has an inner diameter of 2 m, an aperture diameter of 500 mm, and 26 100 V, 500 W halogen lamps. Two lamp power supplies are operated in a constant voltage mode and the 13 lamps associated with each power supply are wired in parallel. The electrical configuration is such that every other lamp around the sphere aperture is connected to the same power supply. The 500 mm diameter exit aperture is spaced approximately 5 cm from the plane that intersects the sphere wall using a grooved, cylindrical tube 500 mm in diameter that is painted black. During the intercomparison, a small silicon photodiode with an infrared absorbing filter was fixed to the aperture of the sphere, providing an independent monitor of the stability of the source. The photodiode was not temperature stabilized and the circuit board for the transimpedance amplifier was mounted outside the sphere, just below the aperture.

**Fig. 1:**
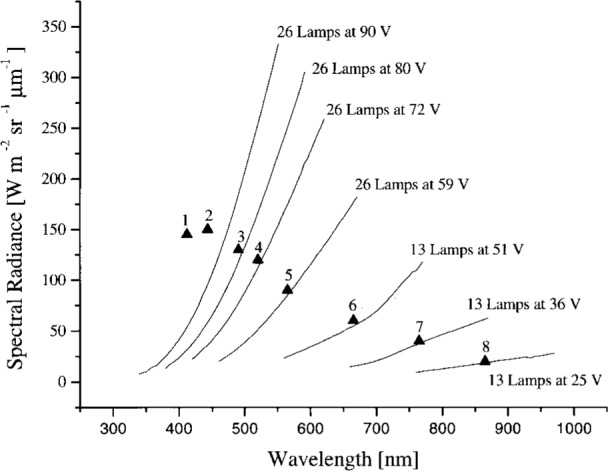
The spectral radiance of the OCTS integrating sphere as measured by NEC in February 1995 for OCTS (ocean) Bands 1 to 8. The solid lines correspond to the measured spectral radiance and the solid triangles correspond to the OCTS ocean calibration spectral radiances. The 26 lamp, 90 V setting was used to calibrate OCTS, even though the actual spectral radiance of the source was about 2.7 (Band 1) and 1.6 (Band 2) times less than the desired calibration radiance. Not much improvement in these ratios would be made with lamp voltages above 90 V but below the maximum value of 100 V.

The sphere was calibrated on February 13–15, 1995 relative to a copper fixed-point standard blackbody source at 1084.62 °C, using a variable-temperature transfer blackbody source operated between 957 °C and 1450 °C and a double grating monochromator. The procedure is described in Ref. [11]. The slit width corresponded to a bandwidth of 5 nm and the step size was 5 nm. For each radiance level required for the calibration of OCTS (for ocean observations), the sphere was operated with different lamp voltages and either 26 or 13 lamps; see Table 2b. The sphere radiance corresponding the lamp configurations in Table 2b are designated Band 1, 2 for the 26 lamp, 90 V setting, Band 3 for the 26 lamp, 80 V setting, and so forth in the remainder of this paper. Figure 1 shows the results of the NEC measurements. The calibration was performed for wavelengths from 100 nm below to 100 nm above the OCTS center wavelengths. The relative standard uncertainty of these data is estimated to be 2 %.

**Fig. 2:**
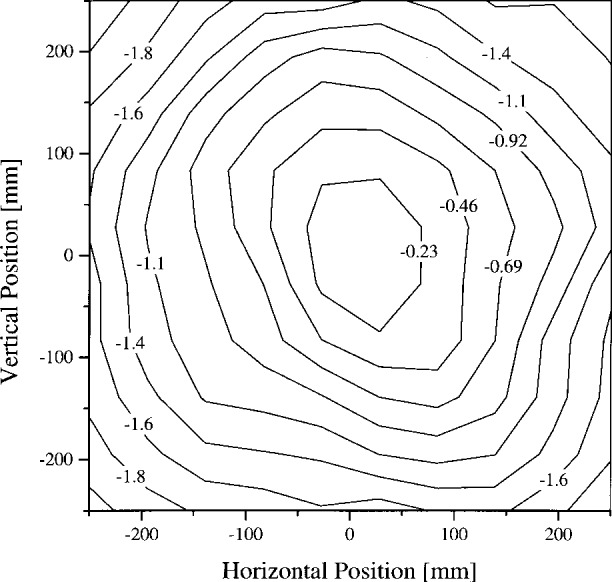
Uniformity of the spectral radiance of the OCTS integrating sphere for the 51 V, 13 lamp configuration (OCTS Band 6) as measured at 650 nm. The relative variation, normalized to the value at the center of the aperture, is shown in units of percent. The average of the normalized radiance over the entire aperture is −0.92 %.

The uniformity of the spectral radiance at the aperture of the sphere was measured every 40 mm on February 16 and 17, 1995. Three radiance levels, corresponding to the lamp configurations for OCTS Band 5, Band 6, and Band 8, were measured. Three filter radiometers that were developed with the support of the Japan Resources Observation System Organization (JAROS) for pre-flight ASTER calibration activities were used [12]. The target area at the aperture of the OCTS sphere was about 4 mm in diameter. The results for the 51 V, 13 lamp configuration (OCTS Band 6) are shown in Fig. 2. The JAROS radiometer with a center wavelength of 650 nm and a bandwidth of 56 nm was used to measure the uniformity of the Band 6 setting. These measurements were normalized to the value at the center of the aperture and the contours correspond to the percent difference from this central value. The radiance is symmetric with azimuthal angle, but decreases with increasing distance from the center of the aperture. For the 59 V, 13 lamp configuration (OCTS Band 5), and the 25 V, 13 lamp configuration (OCTS Band 8), the JAROS radiometers with center wavelengths at 560 nm and 810 nm were used. The spatial coverage was coarser, but the general trend was the same. The variation from center to edge was only about −1.0 % for Band 5 (measured at 560 nm) but this value increased to about −2.2 % for Band 8 (measured at 810 nm).

### 2.2 Transfer Radiometers

#### 2.2.1 NRLM-NASDA Transfer Radiometers

Six filter radiometers with center wavelengths and bandwidths close to those of the OCTS instrument were prepared for the OCTS and POLDER pre-flight calibration activities with the support of NASDA [6]. The center wavelengths and bandwidths for the OCTS radiometers are given in the first two columns of Table 3a, and the properties of the six radiometers are summarized in Table 3b. An objective lens is used to image the target onto a field stop, which is a hole in the center of a turning mirror. Light reflected from the mirror is used for an alignment path. Light passing through the field stop is imaged onto a silicon photodiode after passing through an interference filter. In some cases, the ambient temperature of the detector is monitored so a correction can be applied for the change in responsivity with temperature. The radiometers were calibrated using the NRLM copper fixed-point blackbody source at a distance of 400 mm, which resulted in a target diameter of 3 mm. The spectral responsivity was measured by comparison to a thermopile detector using a lamp and a monochromator as the narrowband source. The linearity with radiant flux was measured over the radiance range from the copper point to the radiance levels measured using the OCTS sphere.

#### 2.2.2 NIST Transfer Radiometer

The SeaWiFS Transfer Radiometer (SXR) was designed and built by NIST for the SeaWiFS Project Office at NASA/GSFC [13]. The SXR uses a camera lens to focus the object at the field stop. Behind the field stop six wedge-shaped mirrors with spherical curvature focus the field stop at six image locations, where individual interference filters and silicon photodiodes are located. The field stop, filters, and detectors are temperature controlled at 26 °C. An on-axis optical system is used to align and focus the SXR. The field-of-view of the SXR is 2.5° and the minimum object distance is 85 cm. The SXR was characterized for radiometric linearity using a beam conjoiner, for relative spectral responsivity using NIST’s calibration facility for detector spectral flux responsivity, and for spatial (field-of-view) responsivity using a small lamp at the object plane and a two-axis translation stage. The radiometric calibration coefficients were determined by measuring an integrating sphere source that had been calibrated on NIST’s calibration facility for spectral radiance and irradiance. The wavelengths and bandwidths, which are close to the SeaWiFS wavelengths, are given in Table 3a and were calculated using a moment analysis that is described in more detail below.

#### 2.2.3 UA Transfer Radiometer

The UA Transfer Radiometer (UAXR) uses a pair of precision apertures to define the solid angle [14]. A filter wheel with eight positions located in front of the first aperture contains seven interference filters with center wavelengths close to the MODIS wavelengths and a shutter for dark signal measurement. A trap detector, consisting of three silicon photodiodes, located behind the second aperture measures the incident flux. The temperature of the UAXR is maintained at 30 °C using a heater circuit. The UAXR is aligned using a cylindrical tube with small holes in either end. This tube, which is the same diameter as the UAXR, fits in the V-block mount during alignment. The vignetted field-of-view is 4.6°. The relative spectral responsivity of the interference filters was measured at the UA’s Optical Science Center using a double monochromator from Optronic Laboratories, Inc. (model OL750-M-D) configured for transmission measurements (accessories 740-73 and OL750-HSD-301). The calibration coefficients were determined using a standard of spectral irradiance. The first aperture in the UAXR was removed, the irradiance responsivity determined, and the radiance responsivity calculated from the aperture geometry. The wavelengths and bandwidths of the seven measurement channels (see Table 3a) were also calculated using a moment analysis.

#### 2.2.4 GSFC Transfer Radiometer

The NASA/GSFC radiometer is a scanning single-grating monochromator that has a 10.2 cm diameter integrating sphere with a known entrance aperture as the collection optic. The monochromator is a Model 746 from Optronic Laboratories, Inc. The system has been termed the 746/Integrating Sphere Irradiance Collector (746/ISIC); a description is in Ref. [15]. The 746/ISIC is calibrated prior to each measurement of an integrating sphere using a 1000 W quartz-halogen standard irradiance lamp that is traceable to NIST. Once the irradiance responsivity is determined, the 746/ISIC measures the irradiance from the integrating sphere. Knowledge of the diameter of the exit aperture of the sphere, the diameter of the entrance aperture of the small sphere on the 746/ISIC, and the distance between the two apertures is used to convert the measured irradiance to the average spectral radiance for the exit aperture of the sphere. At NEC, the 746/ISIC was used from 400 nm to 1100 nm with a bandwidth of about 4.8 nm and a step size of 10 nm. A single grating with 1200 lines per mm blazed at 500 nm was sufficient, and two order sorting filters were used to eliminate second-order effects. The 746/ISIC system is sensitive to scattered radiation, since the field-of-view is the entire hemisphere.

### 2.3 Auxiliary Integrating Spheres

Two small integrating spheres were used to check the stability of the NRLM radiometers and the UAXR following shipment to NEC. NRLM used a 150 mm diameter integrating sphere coated on the inside with barium sulfate. This sphere has four 12 V, 30 W lamps and an exit aperture 30 mm in diameter. The UA used a 152.4 mm diameter Spectralon™^4^ integrating sphere with an exit aperture 50.4 mm in diameter. A single 2.8 A, 30 W lamp was used. The measurement of the NRLM sphere with their radiometers at NEC agreed with the measurements of the sphere at NRLM to within 0.5 %. The measurement of the UA sphere with the UAXR agreed with the results at UA prior to shipment to within 0.2 % except at 868 nm, where the difference was 2.4 %. The SXR was used to measure a sphere source at NIST before and after the experiment at NEC; the average difference was 0.05 % with the largest change at 412 nm (this value decreased by 0.3 %).

## 3. Measurement Procedure

The intercomparison experiment using the OCTS integrating sphere was held in a clean room at NEC Corporation, Yokohama, Japan in February 1995. The OCTS experiment was preceded by similar measurements of the integrating sphere that had been used to calibrate the VNIR instrument that is one of the three radiometers on ASTER [7]. These measurements took place during the first part of the week-long activity. On Monday, February 20, 1995 the participants unpacked their radiometers and performed stability checks using the auxiliary integrating spheres. The ASTER intercomparison experiment lasted until Wednesday afternoon, February 22. The OCTS experiment took 2 days, February 23 and 24. Following the measurements on Friday afternoon, the equipment used by the participants was packed for shipment. Once a day the participants met to discuss the results achieved during the previous set of measurements.

There were several factors that affected the planning of the experiment; some of these issues were not identified until the measurements of the ASTER sphere were underway. First, only 2 days were available for the OCTS measurements. Second, it was not possible to leave any equipment turned on during the night. Third, the various radiometers were designed for different applications, and therefore were not suited to measure all levels of the OCTS sphere with similar accuracy and precision. Fourth, it was not possible to darken the clean room; one source of light was overhead fluorescent lights in a portion of the clean room that was in use by a different project, the other was a set of frosted windows to the outdoors. Fifth, the mechanical configuration of the OCTS integrating sphere required the use of a smaller aperture, 384 mm in diameter.

The OCTS integrating sphere required one hour to stabilize after the lamps had been turned on or adjusted in voltage. The participants’ digital multimeters, transimpedance amplifiers, temperature stabilization systems, and other electronics were turned on at least one hour before the measurements, which, in the case of the digital multimeters, is the minimum recommended time for the instrument to perform within its specified accuracy. The 746/ISIC system is tedious to align and requires additional measurement time to scan the standard irradiance lamp before and after the measurement of the integrating sphere. It was decided to end and begin the rotation of the participants’ radiometers for each radiance level with the 746/ISIC system, reducing the time required to align this system to the sphere aperture. Considering all of these factors, only two of the OCTS sphere configurations (or levels) could be measured once by all participants on each day.

The radiance levels were selected by first restricting the study to the ocean levels. Then, it was noted that none of the filter radiometers could measure near the OCTS Band 4 (520 nm). Bands 5 and 6 have nearly the same center wavelengths as the ASTER VNIR instrument, and could therefore be measured with a set of transfer radiometers developed by NRLM for ASTER. Bands 7 and 8 were assigned low priority because the sensitivity of the UAXR would probably have resulted in reduced measurement precision. The 746/ISIC would also suffer from the decreased radiance, although it has been used at the SIRREX’s to measure even dimmer sources [9]. The four remaining sphere configurations were measured in the morning and afternoon of the two days. On February 23, Band 6 was measured in the morning and Band 3 in the afternoon. On February 24, Band 1, 2 was measured in the morning and Band 5 in the afternoon. The SXR was not designed for radiance levels as high as those used by OCTS, and for the Band 1, 2 configuration the SXR saturated at 442 nm and 662 nm. For the Band 3 configuration, the SXR was nearly in saturation at 662 nm, with signals of about 11.4 V (10 V is the desired maximum signal; at saturation, the output of the SXR is 13 V). The possibility of repeat measurements of the same sphere configuration was not discussed, although this probably would have been a useful experiment.

As mentioned, the clean room was illuminated with overhead fluorescent lighting and a set of outdoor-facing windows. During the calibration of the OCTS sphere by NEC, all overhead lighting was on and the windows were not covered. Because the ASTER and OCTS spheres were located next to each other in the clean room, the effect of the external sources of light on the measured integrating sphere radiance was studied. With the internal lamps turned off, measurements of the OCTS sphere radiance were made using the SXR with all of the overhead lights on and with a subset of lights located directly overhead turned off. The external sources were found to cause a small residual radiance. However, for the 746/ISIC, the external sources produced a larger residual radiance. To reduce this residual radiance, a black cloth tent was constructed between the OCTS integrating sphere exit aperture and the 746/ISIC sphere entrance aperture. Radiance measurements were also made with a 384 mm diameter aperture over the 500 mm exit aperture. This smaller aperture was required for accurate measurements with the 746/ISIC, since the 746/ISIC measurement requires a well-defined configuration factor for the irradiance to radiance transfer. The 500 mm diameter aperture at the end of the 5 cm long cylindrical tube of the same diameter caused vignetting and was a potential source of scattered radiation.

During the measurements on the OCTS sphere, the relative humidity in the clean room was 50 %, and the temperature was 23 °C. The lamp voltages and the output of the silicon photodiode that was mounted on the aperture of the sphere were monitored by recording the signals manually. The configuration of the electrical power grid in Eastern Japan is 100 V, ac, at 50 Hz. In Japan, 100 V ac is across the hot and neutral leads with 50 V ac across the ground and the hot or neutral leads. This is different than the electrical configuration in the United States in which the neutral and ground leads are 110 V ac with respect to the hot lead. Step-up transformers were used to convert to 110 V ac at 50 Hz, and all of the U.S. equipment was connected by bypassing the ground.

The integrating sphere measurement procedure consisted of adjusting the lamp voltages to the desired values, waiting for at least 1 hour for the equipment to stabilize, and aligning the first radiometer to the sphere aperture, recording the signals, and then repeating the sequence with the next participant until the measurements at that sphere level were complete. For the filter radiometers, all measurements were made viewing the central portion of the aperture at normal incidence. The OCTS radiometers that were operated by NRLM were mounted one at a time on a tripod. Only the radiometer that corresponded to the OCTS Band was used. For Bands 5 and 6, the JAROS radiometers that had been developed for the ASTER cross-comparison activities were also used. These radiometers had center wavelengths of 560 nm and 650 nm and bandwidths of 48 nm and 56 nm, respectively (ASTER Bands 1 and 2). They had been used during the prior 3 days for the ASTER intercomparison activity [7]. Measurements were made with the SXR, the UAXR, and the 746/ISIC at measurement wavelengths that cannot directly be compared to the NEC calibration data. Offset signals for the filter radiometer measurements were generally obtained by closing a shutter or lens cap on the radiometer.

## 4. Results

### 4.1 Sources of Bias

The residual radiance of the OCTS sphere was measured using the SXR on February 21 with the 500 mm diameter exit aperture and the internal lamps off. The radiance was measured during daylight hours with all of the overhead fluorescent lights on, those in the half of the room where the intercomparison took place turned off, and also during evening hours in this same configuration. Four of the SXR measurements wavelengths are within 3 nm of the OCTS Bands 1, 2, 3, and 6; the fifth is 17 nm below OCTS Band 5 and the sixth is 10 nm above OCTS Band 7 (see Tables 1 and 3a). Therefore, the ratio of the residual radiance as measured at the SXR wavelengths to the radiance of the OCTS sphere interpolated at the SXR wavelengths (see below) is an indicator of the bias that may exist in the NEC calibration values from the external sources of illumination. The largest observed difference was due to the fluorescent lights that were in the half of the room where the measurements took place. The SXR results indicate that NEC may have overestimated the OCTS sphere radiance by about 0.2 % for OCTS Bands 1, 2, 3, and 6. At 548 nm, the effect was 1.2 %, and at 775 nm, the effect was negligible. However, the actual bias that may exist in the OCTS calibration depends strongly on the spectral output of the fluorescent lights. For example, the Hg emission line at 546.1 nm is within the bandwidth of the 548 nm SXR channel. As for the difference between daylight and evening, it was about 0.01 % for OCTS Bands 1, 2, and 3, 0.04 % for OCTS Band 6, 0.03 % at 548 nm, and 0.06 % at 775 nm. The final results described here correspond to daylight and evening hours, with the fluorescent lights in the section of the clean room used for the intercomparison turned off.

As mentioned earlier, NEC calibrated the sphere and then calibrated OCTS using the full aperture (i.e., 500 mm in diameter) of the integrating sphere. However, the measurements made with the 746/ISIC had to be done with a smaller aperture, 384 mm in diameter, because the 500 mm exit aperture is at the end of the 5 cm long, 500 mm diameter extension tube. The effect of the aperture on the radiance of the sphere was measured with the filter radiometers. These results indicate that the smaller aperture increased the radiance by between 0.1 % and 0.2 %. Both apertures were used for all of the measurements with the SXR and the OCTS radiometers. The UAXR measured the sphere at the Band 1, 2 setting without the 384 mm diameter aperture and with it for the Band 3, 5, and 6 settings.

The sphere appeared to be stable for a given setting of the lamp voltages, based on a study of the Band 1, 2 radiance taken mid-day on February 24 for about 1 h. The SXR and the UAXR were used for this measurement. Viewing the center of the sphere aperture at normal incidence, the signals measured by the SXR one hour apart indicate that the sphere radiance increased by a small amount, 0.08 % at 412 nm and 0.02 % at 775 nm. During this experiment, the UAXR viewed the center of the sphere at an angle and recorded data continuously over the time interval at 443 nm; these data indicate the sphere radiance increased by 0.07 %.

Another measure of the sphere stability was the monitor photodiode on the sphere. For all measurements with the SXR, the signal of the broadband photodiode monitor in the sphere was recorded by entering the values as comments in the SXR data files. The monitor was stable, with a relative standard deviation of about 0.03 %, until the aperture configuration was changed during the middle of the SXR measurement set. After the aperture configuration was changed, the signal on the monitor was not stable, and this was probably caused by the change in the thermal environment of the monitor and its amplifier circuit. The signal on the monitor was about 2 % greater when the 384 mm diameter aperture was in place.

There was no opportunity to verify the repeatability of the measurements of the OCTS sphere by independent studies of any of the bands. However, it is possible to compare the lamp voltages and monitor signals to those during the calibration of the sphere by NEC. The lamp voltages agreed to within the desired values to within 0.01 %. During the NEC calibration, the monitor signal varied by up to ± 0.8 % from the average value, but a greater variation, on the order of 1.5 %, was seen during the intercomparison. This was because the monitor signal changed when the smaller aperture was used (see above). Although written records were kept during these measurements, the status of the aperture plate was not recorded and therefore it is difficult to determine if the magnitude of the monitor signal agreed with the values during the calibration of the OCTS sphere by NEC.

The voltage calibration on two of the digital multimeters, the SeaWiFS Hewlett Packard model 34401A and the UA Hewlett Packard model 3458A, was checked using a precision voltage source from NEC Corporation. The digital multimeters agreed with each other to within the measurement resolution of the 34401A. Over the 500 mV to 5 V range of input voltages, the two meters agreed with the precision voltage source to within 0.008 %; at 100 mV the agreement was within 0.02 %.

One source of bias that is particular to the 746/ISIC measurement is the nonuniformity of the radiance over the sphere aperture. The filter radiometers used in this comparison and the optical system used in the NEC calibration of the sphere measured the radiance at the center of the exit aperture, while the 746/ISIC method measured a spatially-weighted average radiance for the entire aperture. The spatial dependence of the radiance in the OCTS aperture for the Band 6 setting is shown in Fig. 2. Compared to the central value, the average radiance is 0.92 % smaller. The actual bias in the 746/ISIC results requires calculation of the configuration factor with the sphere non-uniformity incorporated in the integrals. Because the spatial uniformity data do not exist in sufficient detail for all sphere levels studied in this comparison, this calculation was not performed.

### 4.2 Analysis for Wavelengths Within the OCTS Spectral Bands

**Fig. 3:**
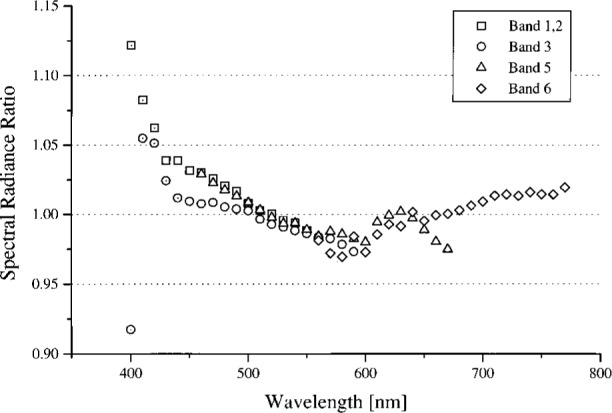
Results of the measurements of the 746/ISIC system of the OCTS integrating sphere for the four bands that were studied during the intercomparison. The 746/ISIC results have been divided by the spectral radiance from the NEC calibration of the sphere just prior to the intercomparison. The symbols with the central dot indicate results where the 746/ISIC results are suspect because of possible contamination by stray light.

The results near the OCTS instrument wavelengths were analyzed in several ways. For the 746/ISIC, the measured spectral radiances were compared to the calibration values from NEC. This was possible because the measurement bandwidths were very similar and the measurement wavelengths overlapped. The spectral radiance of the 746/ISIC divided by the NEC results are plotted in Fig. 3. A small subset of the 746/ISIC results are suspect because of possible contamination by stray light (see Sec. 5) and these values are indicated in Fig. 3.

The average value of the NEC to 746/ISIC radiance for all four bands, excluding the eight suspect values, is 1.000 ± 0.016, where the uncertainty is the standard deviation of the radiance ratios. In Figs. 4a, 5a, 6a, and 7a the spectral radiance of the OCTS integrating sphere as measured by NEC and the 746/ISIC is illustrated. The 746/ISIC data show anomalies near 760 nm and 920 nm to 960 nm; these are consistent features in the 746/ISIC measurements for each of the four sphere levels. The change in the slope of the spectral radiance near 700 nm is also a consistent feature for all four sphere configurations.

For the SXR, the calibration radiance data, *L*_cal_(*λ*), from NEC was used to predict the signal, *S*_pred_, according to the equation

$$S_{\text{pred}}=C\int L_{\text{cal}}(\lambda )r(\lambda )d\lambda ,$$ (1)

where *C* is the calibration constant and *r*(*λ*) the measured relative spectral responsivity of one channel of the SXR.

Equation (1) was solved numerically over the spectral interval corresponding to the NEC calibration. The NEC spectral radiance values were fitted to a model of the form of a polynomial multiplied by Planck’s law of spectral radiance for a blackbody source [16]. The best-fit parameters were used to determine the spectral radiance at the wavelengths required in Eq. (1). The model reproduced the NEC measurements to within ± 0.5 % for most values for Bands 3, 5, and 1, 2; the exceptions were at the shorter wavelengths. For Band 6, the deviations were somewhat larger, about ± 1.0 %. For comparison purposes, a cubic spline was used to interpolate the NEC spectral radiances; at the SXR wavelengths, the results agreed with the blackbody model within ± 0.4 %, except for the 412 nm result for Band 1, 2, where the agreement was within 0.6 %.

The measured signals, *S*_meas_ are compared to the predicted signals in Table 4 as the ratio *S*_meas_/*S*_pred_. The measured signals were corrected for the offset or background voltage and for bias caused by measuring a source that is larger in diameter than the source used to calibrate the SXR. The bias is caused by a small response to sources outside the geometric field-of-view of the SXR, so that the actual radiance is less than the measured radiance. The corrections applied for both the 500 mm and the 384 mm diameter aperture for the SXR measurements at 412 nm, 487 nm, 548 nm, and 662 nm were 0.45 %, 0.12 %, 0.24 %, and 0.52 %, respectively. The uncertainties in these corrections are given in Table 5a, and the method of determining the corrections is given in Ref. [13].

For the OCTS and the ASTER transfer radiometers, the calibration radiance data, *L*_cal_(*λ*), from NEC was used to calculate a band-weighted spectral radiance, *L*_b,pred_, according to

$$L_{b,\text{pred}}=\frac{\int L_{\text{cal}}(\lambda )r(\lambda )d\lambda }{\int r(\lambda )d\lambda },$$ (2)

where *r*(*λ*) is the measured relative spectral responsivity of one of the OCTS or ASTER radiometers. Equation (2) was solved numerically over the spectral interval corresponding to the NEC calibration using linear interpolation in the NEC spectral radiance.

The voltages measured at NEC with the OCTS and ASTER radiometers for the OCTS sphere were used to calculate the measured band-weighted radiance according to

$$L_{b,\text{meas}}=\frac{V_{\text{meas}}}{V_{\text{Cubb}}}\frac{\int L(\lambda ,t_{\text{Cu}})r(\lambda )d\lambda }{\int r(\lambda )d\lambda },$$ (3)

where *V* refers to the output voltage of the radiometer and the subscript refers to the copper fixed-point black-body that was used to calibrate the radiometers at NRLM. The spectral radiance of the reference source, *L*(*λ*, *T*_Cu_), is given by Planck’s law.

The measured band-weighted radiances *L*_b,meas_ are compared to the predicted band-weighted radiances in Table 4 as the ratio *L*_b,meas_/*L*_b,pred_. The measurements were corrected for the offset or background voltage and for bias caused by measuring a source that is larger in diameter than the source used to calibrate the radiometers. For three of the radiometers, the magnitude of the correction was determined by measurements at NEC using the OCTS integrating sphere. A port, 30 mm in diameter, on the back wall and aligned normal to the exit aperture was removed and a dark target was placed behind the sphere port. The radiometers were focused on the center of the exit aperture, but viewed the dark target through the open port. Any remaining net signal was attributed to the size-of-source effect. For the other OCTS or ASTER radiometers, the size-of-source effect was determined at NRLM using a small integrating sphere with a set of apertures, from 6 mm to 50 mm in diameter. The corrections applied for using the 500 mm aperture on the OCTS sphere for the OCTS radiometers at 446 nm, 491 nm, 568 nm, and 670 nm were 1 %, 0.7 %, 0.7 %, and 0.9 % respectively; for the ASTER radiometers at 564 nm and 654 nm the corrections were 0.7 % and 1.1 %, respectively. The uncertainties of these corrections are given in Table 5b.

The results for the UAXR were analyzed using a moment wavelength approach that does not require knowledge of the spectral radiance of the source. This requires an approximation of Eq. (1) of the form

$$L(\lambda_{m,\text{UAXR}})=F_{m}S_{\text{meas}},$$ (4)

where *F*_m_ is a calibration constant and *λ*_m,UAXR_ is the measurement wavelength of one channel of the UAXR. Equation (4) is consistent with Eq. (1) if the spectral responsivity function *r*(*λ*) is given by the Dirac *δ*-function. Since this is never the case, Eq. (4) is an approximation to Eq. (1).

The values for *F*_m_ were determined from the absolute calibration. The measurement wavelength *λ*_m,UAXR_ and the bandwidth (full width at half maximum) Δ*λ*_m,UAXR_ were calculated using:

$$\lambda_{m,\text{UAXR}}=\frac{\int \lambda \;r\left(\lambda \right)\;d\lambda }{\int \;r\left(\lambda \right)\;d\lambda }$$ (5)

and

$$\Delta \lambda_{m,\text{UAXR}}=2.345{\left(\frac{\int r\left(\lambda \right){\left(\lambda -\lambda_{m,\text{UAXR}}\right)}^{2}d\lambda }{\int r\left(\lambda \right)d\lambda }\right)}^{1/2}.$$ (6)

The spectral radiances determined with the UAXR from Eq. (4) and Eq. (5) were normalized to the values *L*_cal_(*λ*_m, UAXR_) and the resulting ratios are given in Table 4. The same modified blackbody fit to the NEC calibration data that was used in the analysis of the SXR data was used for the required interpolation.

The measurement wavelengths and bandwidths for the SXR in Tables 3a and 4 were calculated using a modified form of Eq. (5):

$$\lambda_{m,\text{SXR}}=\frac{\int \lambda L_{\text{sph}}\left(\lambda \right)r\left(\lambda \right)d\lambda }{\int L_{\text{sph}}\left(\lambda \right)r\left(\lambda \right)d\lambda }$$ (7)

and

$$\Delta \lambda_{m,\text{SXR}}=\frac{\int L_{\text{sph}}\left(\lambda \right)r\left(\lambda \right)d\lambda }{L_{\text{sph}}\left(\lambda_{m,\text{SXR}}\right)r\left(\lambda_{m,\text{SXR}}\right)},$$ (8)

where *L*_sph_ corresponded to the spectral radiance of a small integrating sphere source that was used to calibrate the SXR. The SXR bandwidths correspond to the full width of a square profile spectral response model, while those for the UAXR correspond to a Gaussian profile. For the measurement wavelengths for the OCTS and ASTER radiometers reported in Table 4, a form of Eq. (7) was used, but the spectral radiance data corresponded to linear interpolation in the NEC calibration data for the OCTS sphere for the appropriate sphere configuration.

### 4.3 Analysis for Other Wavelengths

The analysis in Sec. 4.2 cannot be used to normalize the participant’s measurements to the NEC values, e.g., the ratios *S*_meas_/*S*_pred_ or *L*_b,meas_/*L*_b,pred_, for spectral regions not measured by NEC. However, it is possible to use the approximate method, Eq. (4), to determine *L*_meas_ for the SXR and the UAXR measurements and compare these values to the spectral radiance determined by the 746/ISIC. To evaluate the error associated with the approximation in Eq. (4), the SXR measurements were compared to the NEC values using the approximate method; the results agreed with the values in Table 4 to within 0.14 %.

For the UAXR, the approximate method was evaluated by estimating other measurement wavelengths using Eq. (7). Source shapes corresponding to the UAXR standard irradiance lamp (F330) and the four levels of the OCTS sphere were considered. For the channels at 488 nm and greater, the results were independent of the method of analysis because all of the calculated wavelengths agreed to within 0.1 nm. At 412.8 nm, the incorporation of a function representing the spectral shape of the source, Eq. (7) vs. Eq. (5), shifts the results to longer wavelengths by as much as 0.46 nm for the sphere at OCTS Band 6. The spectral radiance at this wavelength is about 1 % greater than the radiance at 412.8 nm.

Using the approximate approach, the measured spectral radiance of the OCTS integrating sphere at each of the SXR and UAXR wavelengths was calculated. As in Sec. 4.2, it was necessary to correct the SXR results for the size of the aperture. For the 500 mm and the 384 mm apertures, the correction at 442 nm was 0.14 %. The correction for the 500 mm aperture at 775 nm was 1.22 % and for the 384 mm aperture, the correction was 1.06 %. Once the spectral radiance at all six SXR wavelengths and all seven UAXR wavelengths was determined, the values were normalized by the NEC calibration radiance if the wavelength fell within the spectral interval studied by NEC, or by the result of the 746/ISIC measurements for wavelengths outside these spectral intervals. As before, the NEC calibration values were interpolated at the measurement wavelengths using the modified blackbody model. A cubic spline fit was used to interpolate the 746/ISIC data. These radiance ratios are illustrated in Figs. 4 to 7. The results from Table 4 for the OCTS and ASTER transfer radiometers are also included in these figures.

In summary, the spectral radiance of the four OCTS sphere levels as measured by NEC and the 746/ISIC are shown in Figs. 4a to 7a, the spectral radiance ratios with the NEC measurements as the reference are shown in Figs. 4b to 7b, and the spectral radiance ratios with the 746/ISIC measurements as the reference in Figs. 4c to 7c. The 500 mm aperture was used for the NEC measurements, the 384 mm aperture for the 746/ISIC measurements, and both apertures were used with the filter radiometers.

## 5. Discussion of Results

### 5.1 Spectral Features

The spectral features in the 746/ISIC spectral radiance data that were observed at all four bands could be caused by the spectroradiometer or the source. However, the spectroradiometer is an unlikely cause of the features because they do not correspond to a change in grating or order-sorting filter in the 746/ISIC. These changes cause discontinuities in the spectral irradiance responsivity, resulting in increased sensitivity to mechanical adjustments. Also, in the single case where the NEC calibration data observed the region around 760 nm, the NEC spectral radiance data also exhibit the feature at 760 nm (Fig. 7a). This prompted a radiative transfer calculation of the atmospheric transmittance for the total path from the lamps in the sphere to the spectroradiometers. The path length was estimated to be 40 m to account for scattering within the 2 m sphere and the radiative transfer code LOWTRAN was used. The feature at 760 nm coincides with the ${\;}^{1}\sum_{g}^{+}-{\;}^{3}\sum_{g}^{-}\;$ transition in O_2_. The feature at about 940 nm can be explained by absorption in the 3 ***υ***_OH_ region of H_2_O.

### 5.2 Combined Uncertainties

Comparison of the results for the various instruments requires knowledge of the overall uncertainties for each of the participating radiometers. The uncertainties are estimated by considering the standard deviation of the results at NEC Corporation and any uncertainties associated with the correction of bias.

The relative standard uncertainties for the SXR are given in Table 5a. The absolute calibration is an estimate based on the uncertainty in the radiance of the calibration source and the characterization of the SXR for spectral responsivity, linearity, and stability. The uncertainty associated with the size-of-source correction was based on establishing the range of the correction that is reasonable given the characterization data for the SXR and the measurement conditions at NEC. The uncertainty associated with the method of interpolating the NEC or 746/ISIC radiance data at the SXR measurement wavelengths is also indicated. The measurement uncertainty for the SXR of the OCTS sphere was negligible compared to the other sources of uncertainty and is not included in Table 5a. The relative standard uncertainties were added in quadrature to determine the combined standard uncertainty.

The relative standard uncertainties for the NRLM/NASDA OCTS and the NRLM/JAROS ASTER transfer radiometers are estimated to be 1 % or smaller. This component of uncertainty is associated with the absolute calibration, which is based on a copper fixed-point blackbody source, the uncertainty due to nonlinearity, and accurate measurements of the relative spectral responsivity. Additional components of uncertainty arise from the temporal stability, including shipment, which is estimated to be no larger than 0.5 % based on the measurements with the auxiliary sphere. The change in ambient temperature of up to 5 °C between the calibration laboratory at NRLM and the clean room at NEC could have resulted in up to a 0.1 % change in the radiometer responsivity, based on measurements of the temperature coefficient for the radiometers with the largest sensitivity to temperature. The uncertainty in the correction for the size-of-source is given in Table 5b. The sensitivity to the method of analysis is estimated by comparison of the band-weighted radiances calculated using the NRLM method and the spectral radiance predicted by the modified blackbody model. At the wavelengths for the OCTS and ASTER radiometers given in Table 4, the modified blackbody model radiance is between 0.4 % and 1.5 % greater than the band-weighted radiances. An uncertainty of 0.6 % is taken to represent this effect.

The combined standard uncertainty of the UAXR, 2.2 %, is an estimate based on the uncertainty in the irradiance of the calibration standard and the characterization of the UAXR for spectral responsivity, linearity, and geometry. The measurement uncertainty for the UAXR of the OCTS sphere was negligible compared to this value.

The relative standard uncertainties of the 746/ISIC radiance measurements for the OCTS bands are given in Table 5c. The absolute calibration values include the uncertainty of the reference lamp and its operation, the geometry (area and distance errors), and the overall stability of the 746/ISIC.

According to the manufacturer, the precision of the wavelength drive of the 746/ISIC is 0.1 nm and the accuracy is 1.0 nm. The wavelength accuracy of the 746/ISIC was checked at NEC Corporation using a HeNe alignment laser, but the measured power was not stable due to the randomly polarized output of the laser. This made it difficult to analyze these data. Assuming a standard uncertainty in the wavelength of 1.0 nm, the relative standard uncertainty in the spectral radiance is:

$$\frac{u\left[L\left(\lambda \right)\right]}{L\left(\lambda \right)}=\frac{\lambda }{L\left(\lambda \right)}\frac{dL\left(\lambda \right)}{d\left(\lambda \right)}\frac{u\left(\lambda \right)}{\lambda },$$ (9)

where the symbol *u* represents a standard uncertainty. The results for the four OCTS bands studied are given in Table 5c.

The stray light rejection *η* of the 746/ISIC was measured to be about 2 × 10^−5^ at SIRREX-4 using HeCd and HeNe lasers [15]. The signal *S* at each measurement wavelength is the sum of the signal from radiant flux within the bandpass of the 746/ISIC and the signal that is due to stray light. Because the radiance of the sphere is determined by the ratio of the irradiance from the sphere to that from the standard lamp, the bias caused by stray light depends on the relative spectral shape of the two sources as well as on the stray light rejection of the 746/ISIC. Comparison of the signals reveals that the OCTS sphere had more flux in the red than in the blue when compared to the standard lamp. The ratio of the signals at 1100 nm and 400 nm for the sphere at the Band 1, 2 configuration was 2.8 times greater than the corresponding quantity for the standard lamp. This factor was 5.3 for Band 3, 12 for Band 5, and 16 for Band 6. Given that the desired quantity at each wavelength is $S_{\text{in-band}}^{\text{OCTS}}$ divided by $S_{\text{in-band}}^{\text{LAMP}}$, where $S=S_{\text{in-band}}+S_{\text{stray}}$, the correction factor *ξ* for the spectral radiance at each measurement wavelength is given by

$$\xi =\frac{1-\frac{S_{\text{stray}}^{\text{OCTS}}}{S^{\text{OCTS}}}}{1-\frac{S_{\text{stray}}^{\text{LAMP}}}{S^{\text{LAMP}}}}$$ (10)

The quantity $S_{\text{stray}}^{\text{LAMP}}$ or $S_{\text{stray}}^{\text{OCTS}}$ is independent of wavelength and was estimated according to

$$S_{\text{stray}}\approx \frac{\eta }{\Delta \lambda }\int S\left(\lambda \right)\;d\lambda ,$$ (11)

where Δ*λ* is the bandwidth of the 746/ISIC. According to this calculation, the bias is largest at the shortest measurement wavelengths. However, the magnitude of the bias is sensitive to the value for Δ*λ* and *η*. Instead of using the calculation of the bias to correct the measured spectral radiances, it seems more reasonable to use the results to determine the wavelength interval for the 746/ISIC where the results are suspect because of stray light. These approximate regions are 400 nm to 420 nm for Band 1,2, 400 nm to 440 nm for Band 3, 400 nm to 450 nm for Band 5, and 400 nm to 470 nm for Band 6.

The standard deviation of the measurements with the 746/ISIC was generally negligible compared to the uncertainty in the absolute calibration and the 1 nm uncertainty in the wavelength setting. The exceptions were at the shorter measurement wavelengths for Bands 5 and 6 and for some measurements for Band 3. During the Band 3 study, the temperature of the detection electronics increased, adding noise to the system. A final consideration for the 746/ISIC is the linearity of the system given the range of signals. The results here require linearity over a dynamic range corresponding to 1.5 % to 140 % of the signal from the standard lamp.

### 5.3 Comparison of Results

The agreement between NEC and 746/ISIC is indicated in Fig. 3. The majority of the results are within the combined uncertainty of the 746/ISIC measurements and the NEC calibration data for the OCTS integrating sphere. The approximate 0.2 % bias caused by the 384 mm diameter aperture and the up to 1 % bias caused by the non-uniformity of the radiance in the sphere aperture were not accounted for in Fig. 3. The spectral radiance ratios indicated in Fig. 3 appear to increase with decreasing wavelength for all sphere configurations (except for Band 3 at 400 nm), which may be caused by stray light in the 746/ISIC, if it is assumed that the NEC data are not contaminated by stray light (the double monochromator should have a smaller value for the stray light rejection). Wavelengths for which stray light may be a problem in the 746/ISIC are indicated in Fig. 3 using a dot in the center of the symbol. The scatter from the black cloth that was used to shield the instrument from the room lights could also be affecting the 746/ISIC results. The spectral radiance ratios in Fig. 3 are generally consistent for the four sphere configurations and include a distinctive shape between 550 nm and 650 nm. This may be caused by interpolation in the standard lamp irradiance data, which is provided every 50 nm in this spectral region, or by some feature in the NEC calibration data.

Within the spectral interval measured by NEC, the three types of filter radiometers agree with the NEC calibration data within the combined uncertainties. These results are summarized in Table 4, where the average of all of the results is 1.004 and the standard deviation is 0.01. As explained in Sec. 4.2, the values in Table 4 were determined differently, depending on the participant. For the SXR, the measured signal was compared to the signal predicted from the measurement equation, Eq. (4), using the SXR and NEC calibration data. The modified blackbody model was used to interpolate the NEC data for the evaluation of the integral. For the OCTS and ASTER radiometers, the values in Table 4 are the ratios of band-weighted radiances, using the measurements of the copper fixed-point blackbody source at NRLM and the OCTS sphere, with the spectral radiance from the NEC calibration data, to determine the results. For the UAXR, a simplified measurement equation was used to determine the spectral radiance of the OCTS integrating sphere, and the NEC calibration data were interpolated at the UAXR measurement wavelengths using the modified blackbody model to make the comparison.

The simplified measurement equation, when applied to the SXR data, gives nearly identical results as the integral form of the measurement equation. The simplified equation was applied to all of the SXR data and the results are shown in Figs. 4 to 7, along with the results for the OCTS, ASTER, and UAXR radiometers. If the measurement wavelength was within the spectral interval of the NEC calibrations, the filter radiometer results were normalized using the NEC data. If not, the filter radiometer results were normalized using the 746/ISIC data. The general agreement is within the combined measurement uncertainties.

#### 5.3.1 Bands 1 and 2 (Ocean and Land)

The spectral radiance of the OCTS sphere with all 26 lamps on and 90 V across each bank of 13 lamps is shown in Fig. 4a. The NEC calibration data cover the region from 340 nm to 550 nm, and for this wavelength interval the average ratio of the spectral radiance of the 746/ISIC results to the NEC results is 1.028 ± 0.035 (see Fig. 3). Excluding the results at 400 nm to 420 nm gives 1.015 ± 0.018. The radiance ratios for the filter radiometers that are referenced to NEC are plotted in Fig. 4b. The radiance ratios that are referenced to the 746/ISIC are plotted in Fig. 4c.

#### 5.3.2 Band 3 (Ocean)

The spectral radiance of the OCTS sphere with all 26 lamps on and 80 V across each bank of 13 lamps is shown in Fig. 5a. The NEC calibration data cover the region from 380 nm to 590 nm, and for this wavelength interval the average ratio of the spectral radiance of the 746/ISIC results to the NEC results is 0.9984 ± 0.029 (see Fig. 3). Excluding the results from 400 nm to 440 nm gives 0.9940 ± 0.012. The radiance ratios for the filter radiometers that are referenced to NEC are plotted in Fig. 5b. The radiance ratios that are referenced to the 746/ISIC are plotted in Fig. 5c.

#### 5.3.3 Band 5 (Ocean)

The spectral radiance of the OCTS sphere with all 26 lamps on and 59 V across each bank of 13 lamps is shown in Fig. 6a. The NEC calibration data cover the region from 460 nm to 670 nm, and for this wavelength interval the average ratio of the spectral radiance of the 746/ISIC results to the NEC results is 0.9966 ± 0.0144 (see Fig. 3). The radiance ratios for the filter radiometers that are referenced to NEC are plotted in Fig. 6b. The radiance ratios that are referenced to the 746/ISIC are plotted in Fig. 6c, but results at wavelengths where the 746/ISIC may be contaminated by stray light are not shown.

#### 5.3.4 Band 6 (Ocean)

The spectral radiance of the OCTS sphere with 13 lamps on and 51 V across the odd set of 13 lamps is shown in Fig. 7a. The NEC calibration data cover the region from 560 nm to 770 nm, and for this wavelength interval the average ratio of the spectral radiance of the 746/ISIC results to the NEC results is 0.9986 ± 0.0156. The radiance ratios for the filter radiometers that are referenced to NEC are plotted in Fig. 7b. The radiance ratios that are referenced to the 746/ISIC are plotted in Fig. 7c, but results at wavelengths where the 746/ISIC may be contaminated by stray light are not shown.

Comparison of the figures reveals a degree of consistency for the various channels of the SXR or the UAXR with respect to NEC or the 746/ISIC for the four sphere configurations. This consistency is even more pronounced if the UAXR results for Band 6 are not considered. For example, at 775 nm the ratio of the SXR to the 746/ISIC measurement is 0.991 ± 0.004 for Bands 1, 2, 3, 5, and 6. At 487 nm and 548 nm, the ratio of the SXR to the NEC values is also consistent for the four different sphere configurations. At 868 nm the ratio of the UAXR to the 746/ISIC measurement is 0.994 ± 0.002 for Bands 1, 2, 3, and 5, and the results for these three sphere configurations are also consistent when compared to the NEC data. This indicates that small biases may exist, for example, due to from interpolation of the standard irradiance or radiance data, the determination of the size-of-source correction, or quantities that affect that influence the assignment of calibration factors.

## 6. Conclusions

### 6.1 Measurement Results

The 746/ISIC results, when compared to the NEC calibration data, vary between −2.7 % and 3.9 % for all four sphere configurations if the spectral regions that may be contaminated by stray light are excluded (Sec. 5.2). The average of these normalized 746/ISIC results is close to zero with a relative standard deviation of 1.5 %. The filter radiometer results that are near the channels of the OCTS satellite, when compared to the NEC calibration data, differ by between +1.9 % and −1.9 %, with most results within ± 1 % (Table 4). The average of these results for all four sphere configurations and both the 500 mm or the 384 mm diameter aperture indicate that the filter radiometers are 0.4 % high when compared to NEC, with a relative standard deviation of 1 %. When the 746/ISIC results are interpolated at the wavelengths of the filter radiometers and used to normalize the filter radiometer measurements, the average is nearly unity and the relative standard deviation is 1.8 %. Again, measurements at wavelengths that may be contaminated by stray light in the 746/ISIC have been excluded from the calculation. Therefore, within the combined uncertainties, the NEC calibration of the OCTS integrating sphere agree with the four transfer radiometers near the OCTS wavelengths and the four transfer radiometers agree with each other at other wavelengths. These results demonstrate that it is possible to build and calibrate accurate and portable transfer radiometers and deploy them in the field with good results. The results also demonstrate that the independent methods of realizing spectral radiance described in this paper are in agreement.

Comparison measurements were made for the ASTER integrating sphere just prior to this work [7], where the SXR, the UAXR, the 746/ISIC, and the three ASTER/NRLM radiometers were used to measure the spectral radiance of this sphere. The results are similar to those reported here, although for the ASTER comparison it appeared that the NEC data were somewhat smaller than the results achieved with the portable radiometers. The poorest agreement was in the region from 460 nm to about 510 nm for the brightest sphere level studied. In this case, the SXR, UAXR, and the 746/ISIC results were between 2 % and 4 % greater than the NEC results. In April 1994, the OCTS integrating sphere was measured using the OCTS/NRLM radiometers and POLDER/CNES transfer radiometers [6]. Bands 1, 2, 3, and 5 to 8 were studied. The difference between the OCTS/NRLM radiometers and the NEC values was less than ± 3 % and the difference between the OCTS/NRLM and the POLDER/CNES radiometers was less than ± 4 %. The results reported here are similar, and indicate that the spectral radiance of the OCTS integrating sphere at the time of the calibration of the OCTS satellite by NEC was probably determined within the stated relative standard uncertainty of 3 %.

### 6.2 Recommendations

As in all field experiments, unexpected issues were found to be significant and solutions were found by joint cooperation among the participants. A pre-comparison visit to NEC would have provided valuable information. For example, the power supply from GSFC for operation of the standard irradiance lamp failed and NEC lent a suitable supply for the duration of the experiment. The participants would normally have left the electronic equipment turned on for the entire week, but this was not possible for reasons of security and safety. Instead, NEC personnel arrived by 7:00 am in order to begin the experiment. The difference in the configuration of the power grid in Japan as compared to the United States did not seem to compromise the results. The problem of the external sources of light was solved by turning off some of the overhead lights and constructing a tent around the 746/ISIC experiment. The problem of the configuration of the exit aperture of the sphere required the 384 mm diameter aperture for the 746/ISIC measurements, which fortunately NEC had available.

The participants were able to provide preliminary results immediately after the measurements were made, and it was very useful to discuss these results. Future calibrations and intercomparison experiments should be held in a large room that can be made dark. More information on the uncertainties would have been valuable. The 746/ISIC is difficult to align in its current configuration, but the addition of suitable mounting tables and alignment fixtures would improve the procedure and reduce the measurement time. Wavelength calibrations standards other than the HeNe alignment laser should be included with the 746/ISIC, and the wavelength accuracy should be verified during the experiment. The participants should keep careful, complete records, and the host institution should provide each group with chronological data regarding the sphere operation (lamp voltages, signals from monitor photodiodes) and relevant environmental conditions. Finally, data reduction algorithms that address the comparison of results from different filter- and grating-based radiometers should be developed.

The comparison of the results of the transfer radiometers to the NEC data was limited by the extent of their spectral coverage. For future intercomparisons, the calibration of the common source by the host institute should be extended over all the wavelengths to be studied by the visiting radiometers. The general procedure of varying the voltage in the lamps that are in the OCTS integrating sphere to achieve desired spectral radiances results in different spectral shapes for each sphere configuration. More importantly, the resulting bias in the OCTS calibration coefficients, which is from small but finite radiance responsivity at wavelengths outside the OCTS bandwidths, is difficult to determine without a measurement of the spectral radiance of the sphere at each setting for wavelengths from 400 nm to 1100 nm. That is, the spectral shape of the calibration source was very different for the calibration of each OCTS Band, but when in orbit, the OCTS sensor will see a source of the same relative spectral shape at the same time. The practice of operating the lamps at 30 % of the nominal operating voltage appears to be satisfactory, but this is not a common mode of operation in the United States as it may actually shorten the operating life. An alternative method would be to operate the lamps at the same voltage or current, and hence the same spectral shape, but control the radiance of the sphere by varying the number of lamps operated.

## Figures and Tables

**Fig. 4a:**
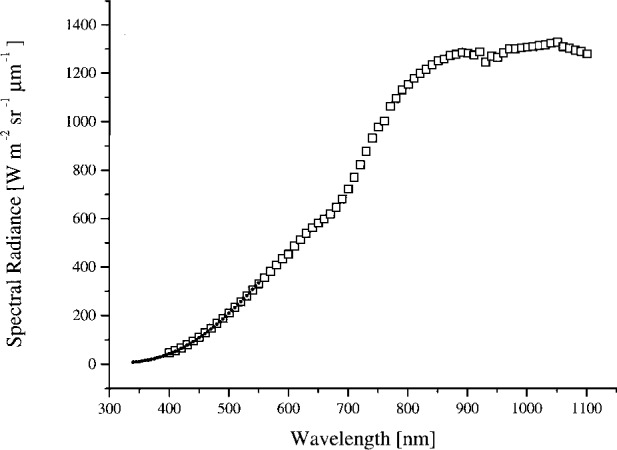
The spectral radiance of the OCTS integrating sphere for the Band 1, 2 lamp configuration as measured by NEC Corporation (small solid circles) and GSFC’s 746/ISIC transfer radiometer (open squares).

**Fig. 4b:**
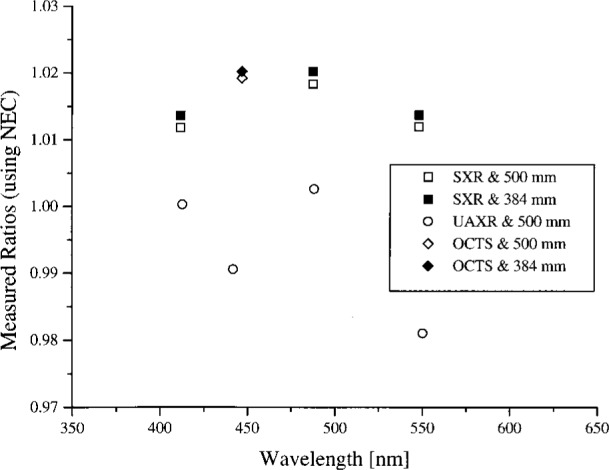
Spectral radiance (SXR and UAXR) or signal (OCTS) ratios as a function of wavelength for the OCTS integrating sphere for the Band 1, 2 lamp configuration as measured by NEC Corporation, NIST, NRLM, and UA. The spectral interval of the NEC calibration was from 340 nm to 550 nm. The NEC measurements were taken with the 500 mm aperture, and both the 500 mm (open symbols) and the 384 mm (solid symbols) were used with the filter radiometers. The filter radiometer results were normalized using the modified black-body model to the NEC calibration data. The square, circle, and diamond symbols denote the results with the SXR, the UAXR, and the OCTS transfer radiometers.

**Fig. 4c:**
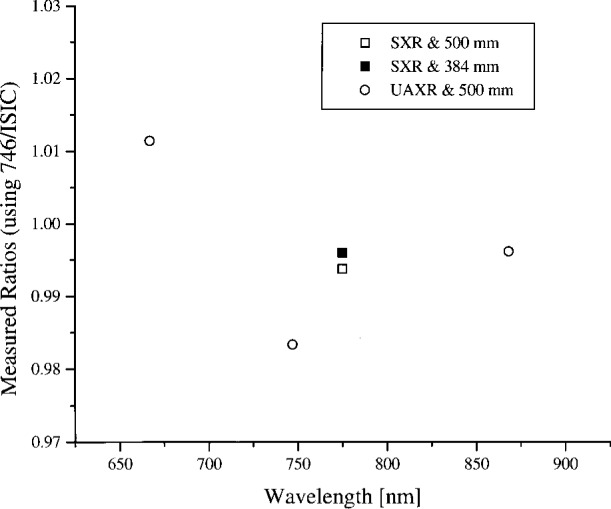
Spectral radiance ratios as a function of wavelength for the OCTS integrating sphere for the Band 1, 2 lamp configuration as measured by GSFC, NIST, and UA. The GSFC 756/ISIC measurements were taken with the 384 mm aperture, and both the 500 mm (open symbols) and the 384 mm (solid symbols) were used with the filter radiometers. The filter radiometer results were normalized to the 746/ISIC data using a spline interpolation. The symbols are as in Fig. 4b.

**Fig. 5a:**
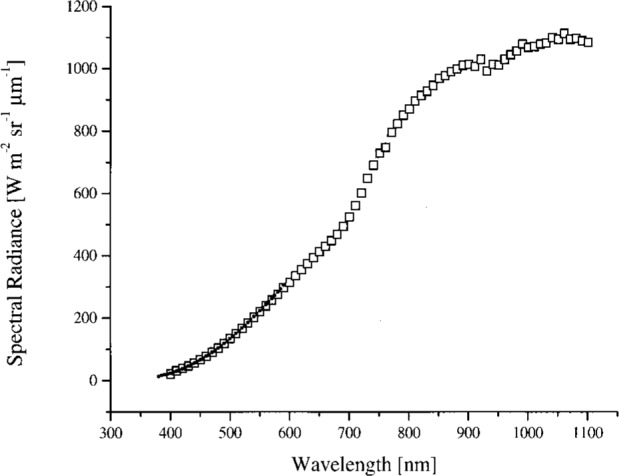
The spectral radiance of the OCTS integrating sphere for the Band 3 lamp configuration as measured by NEC Corporation (small solid circles) and GSFC’s 746/ISIC transfer radiometer (open squares).

**Fig. 5b:**
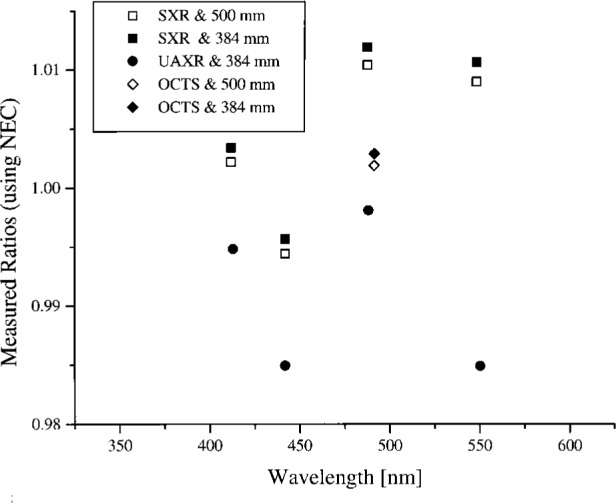
Spectral radiance (SXR and UAXR) or signal (OCTS) ratios as a function of wavelength for the OCTS integrating sphere for the Band 3 lamp configuration as measured by NEC Corporation, NIST, NRLM, and UA. The spectral interval of the NEC calibration was from 380 nm to 590 nm. The NEC measurements were taken with the 500 mm aperture, and both the 500 mm (open symbols) and the 384 mm (solid symbols) were used with the filter radiometers. The filter radiometer results were normalized to the NEC calibration data using the modified blackbody model. The square, circle, and diamond symbols denote the results with the SXR, the UAXR, and the OCTS transfer radiometers.

**Fig. 5c:**
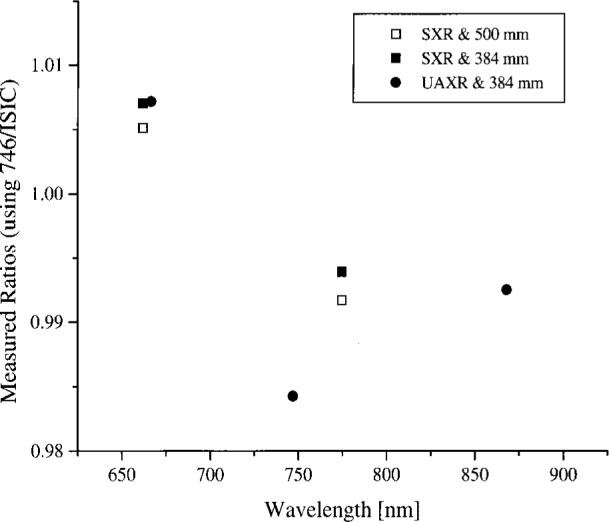
Spectral radiance ratios as a function of wavelength for the OCTS integrating sphere for the Band 3 lamp configuration as measured by GSFC, NIST, and UA. The GSFC 756/ISIC measurements were taken with the 384 mm aperture, and both the 500 mm (open symbols) and the 384 mm (solid symbols) were used with the filter radiometers. The filter radiometer results were normalized to the 746/ISIC data using a spline interpolation. The symbols are as in Fig. 5b.

**Fig. 6a:**
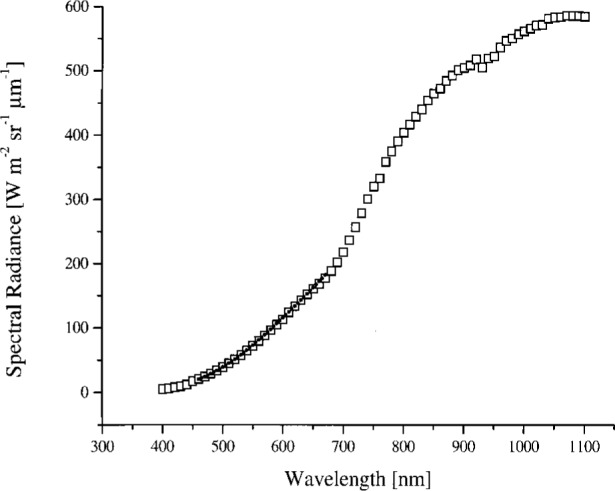
The spectral radiance of the OCTS integrating sphere for the Band 5 lamp configuration as measured by NEC Corporation (small solid circles) and GSFC’s 746/ISIC transfer radiometer (open squares).

**Fig. 6b:**
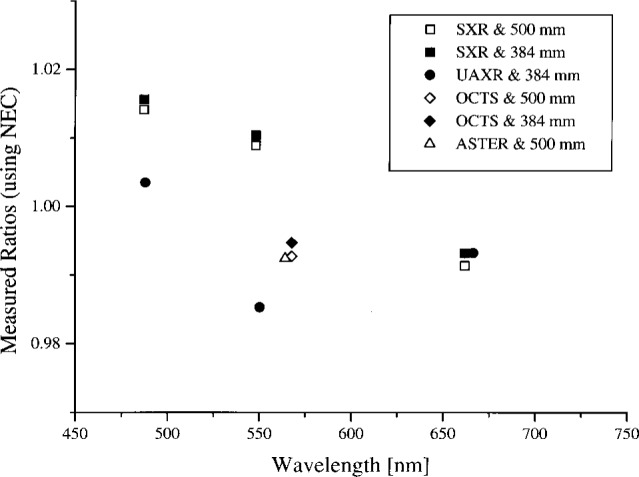
Spectral radiance (SXR and UAXR) or signal (OCTS and ASTER) ratios as a function of wavelength for the OCTS integrating sphere for the Band 5 lamp configuration as measured by NEC Corporation, NIST, NRLM, and UA. The spectral interval of the NEC calibration was from 460 nm to 670 nm. The NEC measurements were taken with the 500 mm aperture, and both the 500 mm (open symbols) and the 384 mm (solid symbols) were used with the filter radiometers. The filter radiometer results were normalized to the NEC calibration data using the modified blackbody model. The square, circle, diamond, and triangle symbols denote the results with the SXR, the UAXR, the OCTS, and the ASTER transfer radiometers.

**Fig. 6c:**
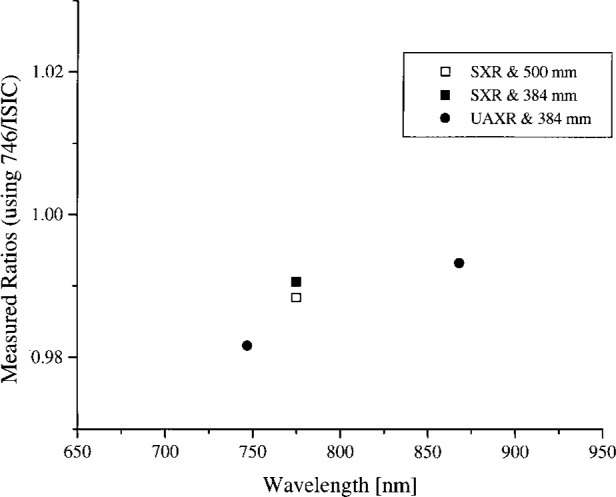
Spectral radiance ratios as a function of wavelength for the OCTS integrating sphere for the Band 5 lamp configuration as measured by GSFC, NIST, and UA. The GSFC 756/ISIC measurements were taken with the 384 mm aperture, and both the 500 mm (open symbols) and the 384 mm (solid symbols) were used with the filter radiometers. The filter radiometer results were normalized to the 746/ISIC data using a spline interpolation. No results are indicated for wavelengths where the 746/ISIC results are suspect because of possible contamination by stray light. The symbols are as in Fig. 6b.

**Fig. 7a:**
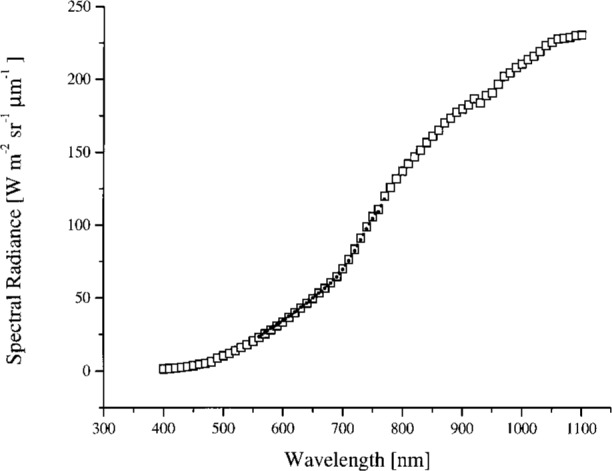
The spectral radiance of the OCTS integrating sphere for the Band 6 lamp configuration as measured by NEC Corporation (small solid circles) and GSFC’s 746/ISIC transfer radiometer (open squares).

**Fig. 7b:**
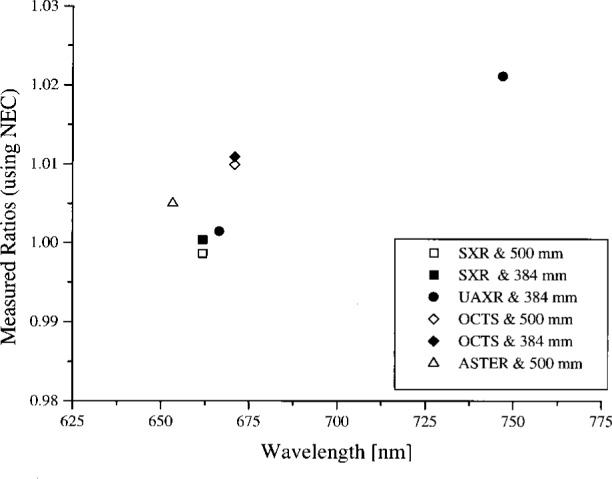
Spectral radiance (SXR and UAXR) or signal (OCTS and ASTER) ratios as a function of wavelength for the OCTS integrating sphere for the Band 6 lamp configuration as measured by NEC Corporation, NIST, NRLM, and UA. The spectral interval of the NEC calibration was from 560 nm to 770 nm. The NEC measurements were taken with the 500 mm aperture, and both the 500 mm (open symbols) and the 384 mm (solid symbols) were used with the filter radiometers. The filter radiometer results were normalized to the NEC calibration data using the modified blackbody model. The square, circle, diamond, and triangle symbols denote the results with the SXR, the UAXR, the OCTS, and the ASTER transfer radiometers.

**Fig. 7c:**
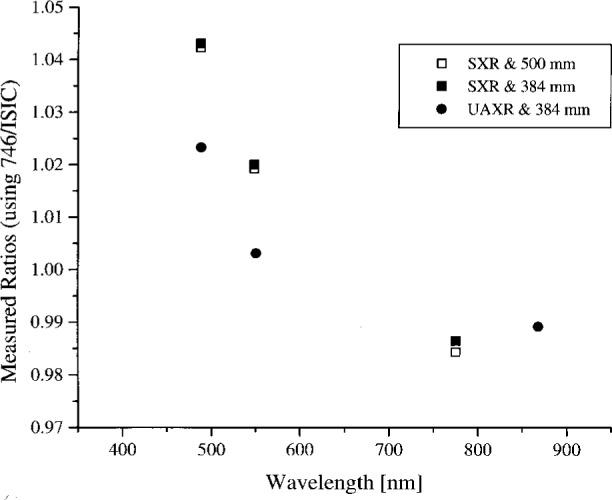
Spectral radiance ratios as a function of wavelength for the OCTS integrating sphere for the Band 6 lamp configuration as measured by GSFC, NIST, and UA. The GSFC 756/ISIC measurements were taken with the 384 mm aperture, and both the 500 mm (open symbols) and the 384 mm (solid symbols) were used with the filter radiometers. The filter radiometer results were normalized to the 746/ISIC data using a spline interpolation. No results are indicated for wavelengths where the 746/ISIC results are suspect because of possible contamination by stray light. The symbols are as in Fig. 7b.

**Table 1:** Center wavelengths, bandwidths, and approximate spectral radiance values during radiometric calibration for the OCTS instrument in the visible and near infrared spectral region

Band number	Center wavelength (nm)	Bandwidth (nm)	Spectral radiance (calibration of OCTS) (W m^2^ sr^−1^ µm^−1^)	
			Ocean	Land
1	412	20	145	145
2	443	20	150	150
3	490	20	130	250
4	520	20	120	290
5	565	20	90	310
6	665	20	60	270
7	765	40	40	230
8	865	40	20	200

**Table 2a:** Specifications of the ADEOS OCTS integrating sphere source

Inner diameter	2 m
Aperture diameter	500 mm
Coating on inner wall	Barium sulfate
Lamp configuration	
Number	26
Maximum voltage (per lamp)	100 V, dc
Input power at maximum voltage	500 W
Power Supply	
Output	0 to 100 V, dc, variable
Relative standard uncertainty (voltage)	0.01 %
Thermal cooling	Air circulation with fans
Monitor detector	Silicon photodiode with infrared blocking filter

**Table 2b:** Parameters of the OCTS sphere during the calibration of the sphere in February 1995, which correspond to the settings used during the calibration of the OCTS instrument for radiance levels corresponding to images of the Earth’s oceans from space

OCTS band number	Number of lamps turned on	Lamp voltage	Temperature of calibration blackbody
		(V)	(K)
1 and 2	26	90.0	1723
3	26	80.0	1645
4	26	72.0	1645
5	26	59.0	1645
6	13	51.0	1230
7	13	36.0	1230
8	13	25.0	1230

**Table 3a:** Wavelengths and bandwidths of the filter radiometers that were used for the measurement of the OCTS integrating sphere in February 1995. The specifications for the NRLM/JAROS/ASTER filter radiometers are given in Ref. [7]

NRLM/NASDA/OCTS		NIST/NASA/SeaWiFs		UA/NASA/MODIS	
Wavelength (nm)	Bandwidth (nm)	Wavelength (nm)	Bandwidth (nm)	Wavelength (nm)	Bandwidth (nm)
445	19	411.5	10.8	412.8	14.9
490	20	441.6	10.3	441.8	11.8
567	24	487.1	10.6	488.0	9.6
670	18	548.0	10.4	550.3	9.9
764	37	661.8	9.57	666.6	9.8
864	42	774.8	11.6	746.9	10.6
				868.1	14.0

Note: The NRLM wavelengths are center values calculated from the 50 % response points and the bandwidths are full-width at half maximum values. The NIST and UA wavelengths and bandwidths are the result of a moment analysis; see Sec. 4.

**Table 3b:** Comparison of the characteristics of the four transfer radiometers that were used to measure the OCTS integrating sphere

	NRLM NASDA/OCTS	NIST SXR	UA UAXR	NASA/GSFC 746/ISIC
Quantity	6	1	1	1
Type (quantity)	Filter (6)	Filter (6)	Filter (7)	Grating (1)
Photodiode (quantity)	Silicon (6)	Silicon (6)	Silicon (3, trap (configuration)	Silicon (1)
Target diameter (mm)	4	45	76	384
Measurement distance (mm)	500	900	900	535
Calibration method	Copper fixed-point blackbody	Radiance standard (sphere)	Irradiance standard lamp (F330)	Irradiance standard lamp (F315)
Saturation radiance^a^ (W m^−2^ sr^−1^ µm^−1^)	5.5 × 10^6^ (443 nm)	67 (442 nm)	≈ 4700 (442 nm)	
	3.9 × 10^6^ (490 nm)	408 (487 nm)	≈ 4600 (487 nm)	
	2.7 × 10^6^ (670 nm)	385 (662 nm)	≈ 3200 (667 nm)	

a Saturation radiance is somewhat arbitrary for the 746/ISIC since the signal can be reduced using several techniques, such as increasing the distance to the sphere under study or decreasing the slit width.

**Table 4:** Comparison of the filter radiometers to the NEC calibration data for the OCTS integrating sphere using the calibration and responsivity data for the filter radiometers. Results for the filter radiometers are for the channel that is near the mid-range of the spectral interval measured for the OCTS integrating sphere. The methods used to determine the wavelengths are described in the text

Radiometer	OCTS band	*X*_meas_/*X*_pred_ 500 mm	*X*_meas_/*X*_pred_ 384 mm	Wavelength [nm]
SXR	1	1.0104	1.0122	411.5
UAXR	1	1.0003		412.8
UAXR	2	0.9906		441.8
NRLM/OCTS	2	1.0192	1.0202	446.4
SXR	3	1.0105	1.0120	487.1
UAXR	3	0.9981		488.0
NRLM/OCTS	3	1.0019	1.0029	491.1
SXR	5	1.0090	1.0105	548.0
UAXR	5	0.9853		550.3
NRLM/ASTER	5	0.9924		564.1
NRLM/OCTS	5	0.9927	0.9947	567.7
NRLM/ASTER	6	1.0050		653.4
SXR	6	0.9984	1.0001	661.8
UAXR	6	1.0014		666.6
NRLM/OCTS	6	1.0099	1.0109	670.9

Note: The quantity *X*_meas_/*X*_pred_ is equal to *S*_meas_/*S*_pred_ for the SXR, *L*_b,meas_/*L*_b,pred_ for the OCTS and ASTER radiometers, and *L*_meas_(*λ*_m_,UAXR)/*L*_cal_(*λ*_m,UAXR_) for the UAXR.

**Table 5a:** Relative standard uncertainties in the SXR as a function of wavelength

Wavelength	Absolute calibration	Size-of-source correction	Interpolation model	Combined uncertainty
(nm)	(%)	(%)	(%)	(%)
411.5	1.5	0.4	0.5	1.6
441.6	1.5	0.3	0.3	1.6
487.1	1.5	0.3	0.3	1.6
548.0	1.5	0.3	0.3	1.6
661.8	1.5	0.3	0.3	1.6
774.8	1.5	0.1	0.3	1.5

**Table 5b:** Relative standard uncertainties for the OCTS or ASTER radiometers as a function of wavelength

Wavelength	Absolute calibration	Size-of-source correction	Stability	Analysis model	Combined uncertainty
(nm)	(%)	(%)	(%)	(%)	(%)
446.4	1.0	1.0	0.5	0.6	1.6
491.1	1.0	0.3	0.5	0.6	1.3
567.7	1.0	0.3	0.5	0.6	1.3
670.9	1.0	1.0	0.5	0.6	1.6
564.1	1.0	0.3	0.5	0.6	1.3
653.4	1.0	0.5	0.5	0.6	1.4

**Table 5c:** Relative standard uncertainties in the 746/ISIC as a function of wavelength and measurement condition

Wavelength and band	Absolute calibration	Wavelength uncertainty	Stray light^a^	Measurement uncertainty	Combined uncertainty
(nm)	(%)	(%)	(%)	(%)	(%)
412 Band 1	1.4	1.8	6.1	0.4	6.5
443 Band2	1.3	1.5	1.5	0.2	2.5
490 Band 3	1.3	1.2	0.56	0.2	1.9
565 Band 5	1.2	0.96	0.34	0.2	1.6
665 Band 6	1.2	0.63	0.12	0.2	1.5

a These values are the estimated bias from stray light, and are used here to approximate the uncertainty caused by stray light.

## Footnotes

1 Certain commercial equipment, instruments, or materials are identified in this paper to foster understanding. Such identification does not imply recommendation or endorsement by the National Institute of Standards and Technology, nor does it imply that the materials or equipment identified are necessarily the best available for the purpose.

2 A complete list of acronyms is given in Appendix A.

3 Formerly Santa Barbara Research Center, a subsidiary of Hughes.

4 “Spectralon” is a registered trademark of Labsphere, Inc., North Sutton, New Hampshire.

## 7. Appendix A. List of Acronyms

ADEOS: Advanced Earth Observing Satellite
ASTER: Advanced Spaceborne Thermal Emission and Reflection Radiometer
CNES: Centre Nationale d’Etudes Spatiales
EOS: Earth Observing System
GSFC: Goddard Space Flight Center
ISIC: Integrating Sphere Irradiance Collector
JAROS: Japan Resources Observation System Organization
JERS-1: Japanese Earth Resources Satellite-1
MODIS: Moderate Resolution Imaging Spectroradiometer
NASA: National Aeronautics and Space Administration
NASDA: National Space Development Agency (Japan)
NEC: not an acronym
NIST: National Institute of Standards and Technology
NRLM: National Research Laboratory of Metrology
OCTS: Ocean Color Temperature Scanner
OPS: Optical Sensor
POLDER: Polarization and Directionality of Earth’s Reflectances
SBRS: Santa Barbara Remote Sensing
SeaWiFS: Sea-viewing Wide Field-of-view Sensor
SIRREX: SeaWiFS Intercalibration Round-Robin Experiment
SXR: SeaWiFS Transfer Radiometer
UA: University of Arizona
UAXR: UA Transfer Radiometer
VNIR: Visible and Near-Infrared Radiometer

## References

[1] Hooker SB, McClain CR, Holmes A (1993). Ocean Color Imaging: CZCS to SeaWiFS. Marine Tech Soc J.

[2] Barnes WL, Salomonson VV (1993). MODIS: A Global Imaging Spectroradiometer for the Earth Observing System. Crit Rev Opt Sci Technol.

[3] Corell R, Harris R (1996). Our Changing Planet, The FY 1997 US Global Change Research Program, a report by the Subcommittee on Global Change Research.

[4] Tanii J, Machida T, Ayada H, Katsuyama Y, Ishida J, Iwasaki N, Tange Y, Miyachi Y, Sato R (1991). Ocean Color and Temperature Scanner (OCTS) for ADEOS.

[5] Deschamps PY, Breon FM, Leroy M, Podaire A, Bricaud A, Buriez JC, Seze G (1994). The POLDER mission: Instrument Characteristics and Scientific Objectives. IEEE Trans Geosc Rem Sens.

[6] Sakuma F, Bret-Dibat T, Sakate H, Ono A, Perbos J, Martinuzzi J-M, Imako K, Oaku H, Moriyama T, Miyachi Y, Tange Y (1995). POLDER-OCTS Preflight Cross-calibration Experiment using Round-robin Radiometers.

[7] Sakuma F, Johnson B Carol, Biggar SF, Butler JJ, Cooper JW, Hiramatsu M, Suzuki K (1996). EOS AM-1 preflight radiometric measurement comparison using the Advanced Spaceborne Thermal Emission and Reflection Radiometer Visible and Near/infrared Integrating Sphere. in Earth Observing System.

[8] Butler JJ, Johnson B Carol (1996). EOS Radiometric Measurement Comparisons at Hughes Santa Barbara Remote Sensing and NASA’s Jet Propulsion Laboratory. The Earth Observer.

[9] Mueller JL, Johnson B Carol, Cromer CL, Hooker SB, McLean JT, Biggar SF, Hooker SB, Fire-stone ER, Acker JG (1996). The Third SeaWiFS Intercalibration Round-robin Experiment, (SIRREX-3), 19–30 September 1994.

[10] Suzuki N, Narimatsu Y, Nagura R, Sakuma F, Ono A (1991). Large Integrating Sphere of Prelaunch Calibration System for Japanese Earth Resources Satellite Optical Sensors.

[11] Sakuma F, Ono A (1993). Radiometric Calibration of the EOS ASTER Instrument. Metrologia.

[12] Sakuma F, Kobayashi M, Ono A (1994). ASTER Round-robin Radiometers for the Preflight Cross-calibration of EOS AM-1 Instruments.

[13] Johnson B Carol, Fowler JB, Cromer CL, Hooker SB, Firestone ER The Sea-WiFS Transfer Radiometer.

[14] Biggar SF, Slater PN (1993). Preflight Cross-calibration Radiometer for EOS AM-1 Platform Visible and Near-IR Sources.

[15] Johnson B Carol, Bruce SS, Early EA, Houston JM, O’Brian TR, Thompson A, Hooker SB, Mueller JL, Hooker SB, Firestone ER (1996). The Fourth SeaWiFS Intercalibration Round-robin Experiment, (SIRREX-4), May 1995.

[16] Saunders RD, Shumaker JB (1973). Optical Radiation Measurements: The 1973 NBS Scale of Spectral Irradiance.

